# Analysis of interleukin-13 receptor alpha2 expression as a prognostic biomarker in surgically resected pancreatic cancer patients

**DOI:** 10.1186/2051-1426-3-S2-P88

**Published:** 2015-11-04

**Authors:** Toshio Fujisawa, Takeshi Shimamura, Bharat H Joshi, Raj K Puri

**Affiliations:** 1 NTT Medical Center Tokyo, Department of Gastroenterology, Tokyo, Japan; 2 Yokohama City University School of Medicine, Department of Oncology, Yokohama, Japan; 3 Center for Biologics Evaluation & Research (CBER)/Food & Drug Administration (FDA), Silver Spring, MD, USA

## Background

IL-13Rα2 is recognized as one of the candidate genes significantly associated with pancreatic cancer risk. Previously, we have demonstrated that IL-13Rα2 is over-expressed in ~70% of human pancreatic cancer samples. We have also shown that IL-13 can mediate invasion and metastasis of human pancreatic cancer cells through IL-13Rα2 both *in vitro* and in an *in vivo* tumor models. Based on these results, we hypothesized that IL-13Rα2 expression in pancreatic cancer may be related to overall survival of subjects following surgical resection.

## Methods

Between 1996 and 2012 we obtained 107 samples from NTT Medical Center Tokyo and 129 samples from Yokohama City University Hospital, Japan. Immunohistochemical staining (IHC) for IL-13Rα2 was performed and the results analyzed independently by each hospital's pathologists. The level of IL-13Rα2 staining intensity (0 to 3+) was used to categorize the specimens as strong (2+ and 3+) or weak expressers (0 and 1+).

## Results

By Kaplan-Meier method, subjects expressing strong IL-13Rα2 on their tumors survived significantly shorter duration compared to those with weak expression (*p* = 0.024). Further analysis demonstrated that the level of IL-13Rα2 expression was inversely correlated with survival time and invasion, but not with tumor staging and histological grade. We are currently examining the correlation between a variety of other clinical factors and IL-13Rα2 expression using Spearman's rank correlation test.

## Conclusions

In summary, our preliminary results suggest that IL-13Rα2 expression has an important role in prognosis and may be critical in overall survival of pancreatic cancer patients.

